# Supplementary material for: Chromothripsis during telomere crisis is independent of NHEJ, and consistent with a replicative origin
Source: Genome Res. 2019 May;29(5):737–49. doi: 10.1101/gr.240705.118 (PMC6499312; doi:10.1101/gr.240705.118)
Supplement: Supplemental Material [file supp_gr.240705.118_Supplemental_file_1.zip › contigs/annotated_contigs/DB116/contig.2.DB116_length_459_mean_cov_3.90849673203.docx]

**DB116_length_459_mean_cov_3.90849673203**

```
ATGAACCACCACCTCCTGGCTAATTTTTTGTATTTTTAGTAGAGACAGGGTTTCACCATGTTGGCCAGGTTGGTCTCCAACTCCTGACC
 >chr10:21734980-21735193 + E=1e-116
TCAAGTGATCTGCTTACCTTGGCCTCTCAAAGTGCTGGGATTACAGGAATGAGCCACCGCACCCAGCCTAGACAATTTTTAATATCAGC

AATTTTTCAACACAGTTCTTTTTCCTTTCCTT|TTC|ATCCTGTACTACATTTGATACTTTGGCCCCAAAAAGTAAGTATTGAAAGTAA
                                 >chr10:21733727-21733976 - E=2e-138
TGGTGGAGGGGGAAATCTCTGTACTCCAGGAATTTACAGTCTGGTTTGGGAGCCAATACTAATAATCGGAAAAGAGTAAAAGACTCTAT

GATTAAGAGCTAAGCCATGTGTTAGAGACTATAAGTATACAGGAAATCAGAGGGTAAAAATCACAGCAGGCTTCATAGATCAGTTAAGA

ACTCAGAGCTTCTAGT
```
